# Histopathological characterization of skin and muscle lesions induced by lionfish (*Pterois volitans*) venom in a murine experimental model

**DOI:** 10.1590/1678-9199-JVATITD-2024-0050

**Published:** 2025-01-17

**Authors:** Cecilia Díaz, Arturo Chang-Castillo, Natalia Ortiz

**Affiliations:** 1 Department of Biochemistry, School of Medicine, University of Costa Rica, San José, Costa Rica.; 2 Clodomiro Picado Institute, Faculty of Microbiology, University of Costa Rica, San José, Costa Rica.

**Keywords:** lionfish, myonecrosis, skin lesion, hyaluronidase, GAPR1, venom

## Abstract

**Background:**

Fish venoms have been poorly characterized and the available information about their composition suggests they are uncomplicated secretions that, combined with epidermal mucus, could induce an inflammatory reaction, excruciating pain, and, in some cases, local tissue injuries.

**Methods:**

In this study, we characterized the 24-hour histopathological effects of lionfish venom in a mouse experimental model by testing the main fractions obtained by size exclusion-HPLC. By partial proteomics analysis, we also correlated these *in vivo* effects with the presence of some potentially toxic venom components.

**Results:**

We observed a strong lesion on the skin and evident necrosis in the skeletal muscle. None of the tissue-damaging effects were induced by the fraction containing cytolysins, membrane pore-forming toxins ubiquitously present in species of scorpionfish, stonefish, and lionfish, among others. On the contrary, injuries were associated with the presence of other components, which have remained practically ignored so far. This is the case of an abundant protein, present in venom, with homology to a Golgi-associated plant pathogenic protein 1-like (GAPR1), which belongs to the same protein superfamily as venom CRISPs and insect allergens.

**Conclusion:**

This GAPR1-like protein and the hyaluronidase are probably responsible for the hemostasis impairment and hemorrhagic lesions observed in mouse skin, whereas muscle injuries can be indirectly caused by a combination of inflammatory and hemorrhagic events. More information is required to establish the components accountable for the myonecrotic effect.

## Background

It is considered that approximately 54% of extant vertebrates are fish and around 7-9% of fish species are thought to be venomous, including members of more than 50 different families [1, 2]. Some fish have very well-developed venom glands (associated with spines or teeth) but others display only groups of venom-producing cells associated with the anterolateral grooves of certain spines [3, 4], making it more difficult to obtain and study their toxic secretions at the transcriptomic level. Moreover, the presence of epidermal mucus and ichthyocrinotoxins [5, 6] complicates the isolation of pure venom compounds and the characterization of the effects of these potential toxins in organs and tissues. 

There are several medical reports of fish envenomation in humans [7-9]. However, the specific pathological effects are only partially described, and no clear causal relationships between the few potential toxins identified so far in these secretions and the reported symptomatology have been completely established. The common denominator event observed in these victims seems to be the presence of a pain-inducing pro-inflammatory reaction, which has been attributed to the combined effect of fish venom compounds and epidermal mucus [5, 10-12].

Scorpaeniformes fish venom, particularly from Synanceiidae (stonefish) and Scorpaenidae (lionfish and scorpionfish), has been the most investigated one due to its medical relevance [8, 11, 13-15]. In experimental animals, the venom of members of this order induces acute inflammatory reactions in muscles, lungs, and other organs, with the presence of interstitial hemorrhage, plasma extravasation, and, in some cases, necrosis, resembling what is observed in the victims of fish encounters [13, 16-18]. 

*Pterois volitans* is a non-native fish that inhabits the Caribbean Sea and western Atlantic Ocean coasts of America. It is considered an ecological danger to the coral reefs because of its invasive behavior [19], and its toxicity for humans has been associated with intense pain, inflammation, and local symptomatology [7, 20]. In the pathological events caused by this fish, an old report [21] indicates that epidermal and dermal alterations could be present, depending on the degree of the injury. These authors report patients with erythematous reactions, blister formation, and dermonecrosis, in the most serious cases. Balasubashini et al. [22] reported bleeding, swelling, congested blood vessels, infiltration of inflammatory cells in mouse vital organs such as the liver, lungs, and kidney, and myofibril degeneration in cardiac muscle. Similarly, after only two hours of intradermal injection of lionfish venom extracts, Sáenz et al. [23] reported an increase in vascular permeability and prominent skin lesions, the latter only being present in mice injected with the venom, and not with epithelial mucus.

The toxicity caused by these venoms has been attributed mostly to hemolysins/cytolysins [24-26], secreted dimeric or oligomeric pore-forming toxins with activity on erythrocytes from some species, and probably in other types of cells [23, 25, 27]. In addition, hyaluronidases, ubiquitous enzymes found in the venoms of most organisms, including fish [28, 29], have been established as accessory components that could facilitate the spreading of toxins into the organism [30]. Some studies, however, suggest that hyaluronidases could participate more actively in the envenoming process [31], since hyaluronic acid and its fragments are involved in inflammation and vascular alterations by regulating leukocyte trafficking and erythrocyte aggregation, interacting with different membrane receptors [32]. Hyaluronic acid is an important component of the extracellular matrix and base membrane. Its degradation could also cause the loss of capillary integrity which could facilitate its rupture and the consequent blood extravasation [33].

It is widely implied that fish venoms are seemingly much less complex than other vertebrate and invertebrate toxic secretions [15, 34, 35], but it does not seem likely that all the effects reported in envenoming victims could be attributed to one type of protein, the cytolysins exclusively. Moreover, there are no clear co-relationships between their proven cytolytic/hemolytic effects and the physiopathology of the envenoming. Thus, more evidence is required to understand the clinical manifestations observed in these human injuries.

In this study, we evaluated the 24-hour *in vivo* histopathological events following the injection of *P. volitans* venom on mouse skin and gastrocnemius muscle, to characterize the possible alterations induced by the compounds present in the main fractions of this secretion. Partial separation of the spine extract components could confirm whether the *in vivo* damage of organs and tissues is caused by the cytolysin-containing fraction or whether other potentially toxic proteins present in the venom could be responsible for the observed injuries.

## Methods

### Fish collecting and preparation of spine extracts

**Figure 1.**
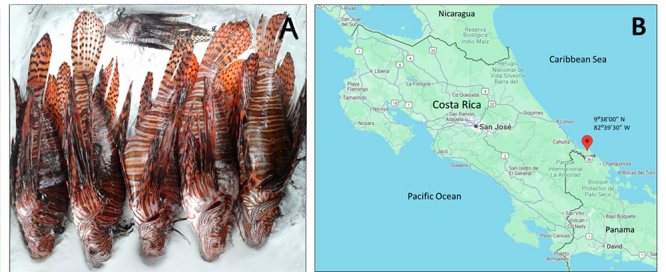
Lionfish and collecting location. **(A)** Lionfish collected specimens, before removing dorsal spines and their utilization for venom extraction and characterization of the *in vivo* effects on mice. (**B)** Map of Costa Rica with the location of the Caribbean coast in the Gandoca-Manzanillo Wildlife Refuge (Province of Limón), where lionfish were collected. The image was modified from Google Maps.

Approximately 10 to 12 adult *Pterois volitans* lionfish specimens of similar sizes (Figure 1 A ) were collected on the Costa Rican Caribbean coasts of Manzanillo Beach (Figure 1 B ), in September-October through the years 2022 and 2024, as part of a local community campaign to decrease the populations of this invasive species, considered an ecological danger to the marine ecosystems [36]. Fishermen from the local community captured and killed the fish by artisanal techniques. Lionfish dorsal spines were cut at the base, placed into cold distilled water, and taken to the laboratory into ice packs. Then, maceration was performed in a ceramic mortar, always on an ice bath, which preserves the viability of the toxins. Samples were centrifuged at 6,500 *g* to eliminate tissue debris and were frozen at -70°C until the experiments were carried out. This study was approved by the Biodiversity Committee of the University of Costa Rica (CBio-46-2022). 

### Fibrinogenolytic activity

Venom fibrinogenolytic activity was evaluated by incubating 20 µg of human fibrinogen (Sigma Chemical Co.) with the extract (2 or 3 µg) in a final 40 µL volume for 3 hours at 37°C. Then, the mixture was analyzed by SDS-PAGE (12%) as described, and the degradation of fibrinogen chains was determined by Coomassie blue R-250 staining of the gels [37]. Protease inhibitors PMSF and EDTA, 10 mM, were utilized to determine whether the fibrinogenolytic activity was associated with serine proteases or metalloproteases, respectively.

### Size-exclusion HPLC for lionfish extract separation 

Venom (2 mg) was separated by size-exclusion chromatography (SEC-HPLC) in a S2000 BioSepSEC column, equilibrated with 0.06M NaCl, and 0.02M sodium phosphate solution (pH 7.2). The first five fractions were collected, dried in a speed vac, and kept at -70°C until testing on mice. Since the utilized column was analytical, we repeated the chromatography ten times and combined the same retention time fractions to have enough protein content to do the *in vivo* assays.

### Electrophoresis and zymography

Venom and size-exclusion chromatography fractions were separated by SDS-PAGE (12%) under non-reducing and reducing conditions (5% 2-mercaptoethanol, at 100°C for 10 min), in a Mini-Protean system (Bio-Rad) at 150 V, and proteins were stained with Coomassie blue R-250.

Zymography for analyzing hyaluronidase activity in the size-exclusion chromatography fractions was determined by the method reported by Cevallos et al. [38] based on SDS-PAGE in a 12% gel containing 0.5 mg/mL hyaluronic acid from rooster comb (Sigma Chemical Co.). The incubation buffer (0.1 M NaCl, 0.1 M sodium phosphate) was adjusted to pH 6.6. Gels were stained with Alcian Blue 8GX (Sigma Chemical Co.) and destained with 5% acetic acid.

Gelatin zymography to determine the proteolytic activity of the size-exclusion chromatography fractions and the semi-purified GAPR1-like protein (see below) was also carried out. Briefly, fractions were subjected to SDS-PAGE on 12% gels containing type A gelatin (Sigma Chemical Co.) at a 0.25 mg/mL concentration. After washing for one hour with 1% Triton X-100, the gel was incubated at 37°C for 16 hours in 50 mM Tris-HCl buffer, pH 8.0, containing 5 mM CaCl_2_, and stained with Coomassie. Blue R-250.

### Direct hemolytic activity

Rabbit blood was collected by ear vein cannulation and erythrocytes were obtained by centrifugation of the citrated blood, previously washed with phosphate-buffered saline and used for the hemolytic assay. Size-exchange chromatography fractions from dorsal spines venom were tested on a 10% erythrocytes suspension and absorbances were read at 545 nm. 

### Mass spectrometry

Separated SDS-PAGE bands - obtained from SEC-HPLC fractions and corresponding to the proteins of interest - were excised from gels and subjected to reduction with dithiothreitol (10 mM) and alkylation with iodoacetamide (50 mM), followed by overnight in-gel digestion with sequencing-grade bovine trypsin (Sigma Chemical Co.) in an automated workstation (Intavis). The resulting proteolytic peptides were extracted with 5% formic acid and analyzed by RP-HPLC-MS/MS as described above, using a shorter gradient with solution B (80% acetonitrile, 0.1% formic acid), as follows: 1-5% B in 1 min, 5-26% B in 25 min, 26-79% B in 4 min, 79-99% B in 1 min, and 99% B in 4 min, for a total time of 35 min [39]. The obtained peptide sequences were manually searched using BLAST (https://blast.ncbi.nlm.nih.gov) to find the most similar proteins in public databases. 

### Purification of GAPR-1-like protein by Benzamidine-Sepharose and RP-HPLC

A small column (2 mL plastic syringe) was prepared with fast-flow Benzamidine-Sepharose (Sigma-Aldrich) and washed with distilled water. Lionfish spine crude extract and size-exclusion HPLC fraction 4, were applied to the column and bound proteins were eluted with 0.1 M glycine (pH 3.0). The sample pH was equilibrated again to 7.2 and proteins were separated by SDS-PAGE (12%) under reducing and non-reducing conditions.

Benzamidine-Sepharose retained samples, which contain the GAPR-1-like protein, were separated by a second chromatographic step through RP-HPLC. Briefly, dried fractions were dissolved in 200 µL of 0.1% trifluoroacetic acid (TFA; solution A) and analyzed by reverse-phase HPLC on a C_18_ column (250 x 4.6 mm, 5 µm particle size; Luna Omega Phenomenex) using an Agilent 1220 chromatograph with monitoring at 215 nm. Elution was performed at 1 mL/min by applying the following gradient with solution B (acetonitrile, containing 0.1% TFA): 0-60% B over 60 min. The purified protein was dried in a speed-vac and identification was performed by mass spectrometry from SDS-PAGE protein bands cut from the gels, previous to the *in vivo* experiment.

### *In vivo* experiments

Animal experiments were conducted following protocols approved by the Institutional Committee for the Use and Care of Animals from Universidad de Costa Rica (CICUA-016-2022).

Groups of four CD-1 mice (18-20 g) were injected intradermally (i.d.) with the lionfish dorsal spine extract and size-exclusion chromatographic fractions of the separated venom, previously dissolved in distilled water. Purified ~34 kDa GAPR-1 protein was also injected i.d. in two other mice. A control group of animals received an identical injection of saline solution. At 24 hours, animals were euthanized by cervical dislocation, and hemorrhage was assessed as described [40]. Skin samples were processed for histological analysis. Slides were stained with hematoxylin and eosin or for the presence of fibrin clots, with Martius, Scarlet, and Blue (M.S.B), according to the kit instructions (DiaPath, Italy).

The myotoxic activity was determined in CD-1 mice (18-20 g) after intramuscular injection of the right gastrocnemius with dorsal spine extract and size-exchange chromatographic fractions dissolved in 100 µL of distilled water. A control group of mice received an identical injection of distilled water. After 24 hours, mice were humanely killed by cervical dislocation, and gastrocnemius was surgically removed and processed for histological analysis [41]. Slides were stained with hematoxylin and eosin.

The effect of the lionfish extract on the hematocrit of CD-1 mice was measured. Briefly, two mice were injected intravenously (i.v.) with 140 µg of venom and after 2 hours, animals were bled from the tail, blood was collected into heparinized capillary tubes and centrifuged to determine the volume of packed red blood cells. Control mice were injected with saline solution under identical conditions. The tubes were also observed for hemolysis. 

## Results

### *In vivo* effects of the venom extract

**Figure 2.**
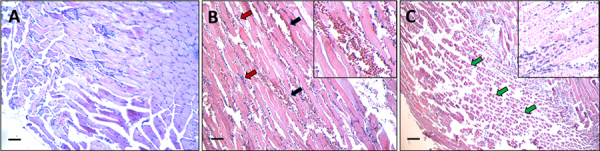
Tissue damage of mice gastrocnemius muscle caused by venom after 24 hours. **(A)** Longitudinal section of control mouse gastrocnemius injected with distilled water. **(B, C)** Longitudinal sections of mouse gastrocnemius injected with venom extract showing alterations such as hemorrhage (black arrows), abundant inflammatory infiltrate (red arrows), and severe myonecrosis (green arrows). Figure insets show an area of amplification of micrographs **B** and **C** that evidences the toxicity provoked by the venom on mouse gastrocnemius. Scale bars represent 100 µm.

Histological analysis shows that the injection of 50 µg of lionfish dorsal spine venom (which also contains epidermal mucus) for 24 hours induces myonecrosis and bleeding in the gastrocnemius of mice. The presence of inflammatory cells in the injected muscle was also evident (Figure 2). 

Moreover, when intravenously injected (140 µg), it slightly decreased the hematocrit value from 39.9 to 30.6 compared to the control, an effect that was not accompanied by *in vivo* red blood cell lysis.

### Venom separation and *in vitro* activities of the fractions

**Figure 3.**
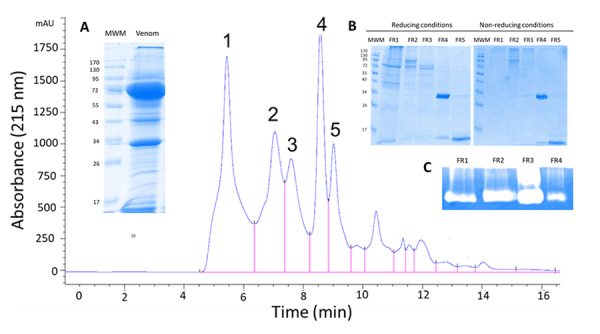
Size exclusion-HPLC of lionfish dorsal spine extract (including epidermal mucus), protein pattern, and hyaluronidase activity. SEC-HPLC chromatogram of the lionfish venom showing the main fractions (numbered from 1 to 5). **(A)** Figure inset: SDS-PAGE (under reducing conditions) of crude spine venom. **(B)** Figure inset: SDS-PAGE (under reducing and non-reducing conditions) of the crude venom extract's first five chromatographic fractions, FR1 to FR5. **(C)** Inset showing a zymography on hyaluronic acid of the first four fractions.

Venom was separated by size exclusion-HPLC into several fractions (Figure 3, inset A), from which the first five were analyzed by SDS-PAGE. Reducing and non-reducing conditions of the samples show differences in the molecular mass of the proteins contained in the fractions, except for the case of fractions 4 and 5, which show no differences in the presence of β-mercaptoethanol. Fractions 4 and 5 contained mainly proteins of ~34 kDa and ~15 kDa, respectively. The other three fractions display several proteins that probably form dimers or oligomers since their masses shift when reducing conditions are applied to the electrophoresis separation (Figure 3, inset B).

Direct hemolytic activity on rabbit erythrocytes was measured in all the fractions to determine the presence of the cytolysins. The only fraction that displayed the activity was fraction 2. *P. volitans* cytolysins are heterodimers of ~160 kDa [26], a molecular weight that could correspond with the size of proteins eluting in fraction 2. 

Hyaluronidase activity was observed in the first four fractions. Still, the strongest effect was observed in fraction 3, in which zymography showed activity at two different molecular weights, indicating that hyaluronidase probably forms homodimers, or heterodimers with other proteins (Figure 3, inset C). The main proteolytic activity on gelatin was detected in the first two fractions, showing several zymography bands displaying this activity (not shown). 

### *In vivo* effects of the venom fractions

When mice were injected with the fractions containing the different proteins of the venom and epidermal mucus, we observed differences in the effects on gastrocnemius muscle and skin at 24 hours.

**Figure 4.**
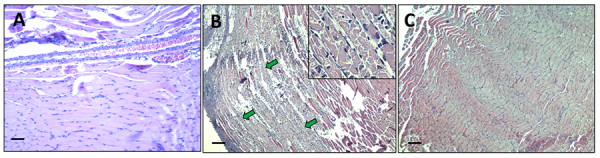
Tissue damage of mice gastrocnemius muscle induced by fraction 1 after 24 hours. **(A)** Longitudinal section of control muscle of mice injected with water. **(B)** Longitudinal section of mouse gastrocnemius injected with fraction 1 displaying severe damage of muscle fibers (green arrows), extensive leukocyte infiltrate, and almost absent hemorrhage. **(C)** In contrast, the mouse gastrocnemius muscle injected with fraction 2 showed no evidence of tissue injury. Figure inset in **B** shows an area of amplification that evidences the myofibrils fragmentation induced by fraction 1 in mouse gastrocnemius. Scale bars represent 100 µm.

Fraction 1**,** which contains the highest molecular weight components and displays proteolytic activity, was able to induce strong myonecrosis whereas fraction 2, which contains the hemolysins present in all Scorpaenidae fish family members (and displays evident proteolytic activity) did not induce apparent injuries on mice muscle (Figure 4). Fraction 1-induced muscle damage was very severe, but contrary to what was observed with the crude venom, almost no hemorrhage was observed in muscle injected with this fraction (Figure 4 B).

**Figure 5.**
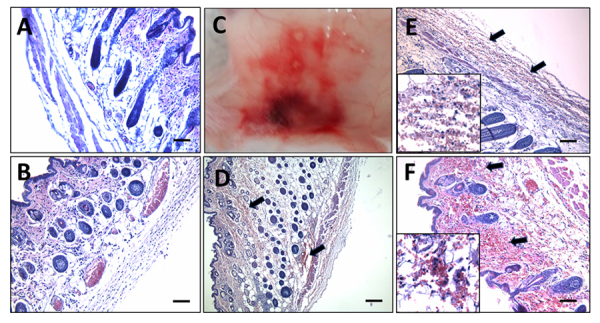
Mouse skin lesions and hemorrhage caused by fractions 3, 4, and 5 after 24 hours. **(A)** Control skin of mice injected i.d. with water. **(B)** Skin of mice injected i.d. with fraction 2, where no hemorrhage was observed, only the presence of inflammatory infiltrate. **(C)** Macroscopic lesion observed in the skin of a mouse injected i.d. with fraction 3, similar to the ones observed with fractions 4 and 5 (not shown). **(D)** Skin of mice injected i.d. with fraction 3, **(E)** fraction 4, and **(F)** fraction 5 showing extensive hemorrhage (black arrows). Figure insets show an area of amplification of micrographs **E** and **F** that evidences the presence of erythrocytes outside the blood vessels and the inflammatory cells in the skin of mice injected with fractions 4 and 5, respectively. Scale bars represent 100 µm.

Fraction 3, which contains the strongest hyaluronidase activity (in two different molecular weight bands, Figure 3 B ), was very toxic on mice skin tissue, inducing a macroscopic lesion, hemorrhage, and an increase in vascular permeability (Figures 5 C and 5D). 

Fraction 4, containing a ~34 kDa very abundant protein with similarity to the Golgi-associated plant pathogenesis-related protein family (GAPR1-like), also induced lesions and hemorrhage on the skin. A similar hemorrhagic effect was observed when fraction 5 was injected, which contains a ~15 kDa protein with similarity also to GAPR1 (Figures 5 C , 5E, and 5F). SDS-PAGE shows that fraction 3 could contain some level of the ~34 kDa protein, which could explain the same effects observed in the skin of mice injected by these fractions (Figure 3 A).

Interestingly, fraction 2, containing hemolysins and displaying hyaluronidase activity, did not induce any evident injury on the skin, except for the presence of leukocyte infiltrate (Figure 5 B ).

### *In vivo* effect of ~34 kDa purified GAPR1-like protein in mice skin

**Figure 6.**
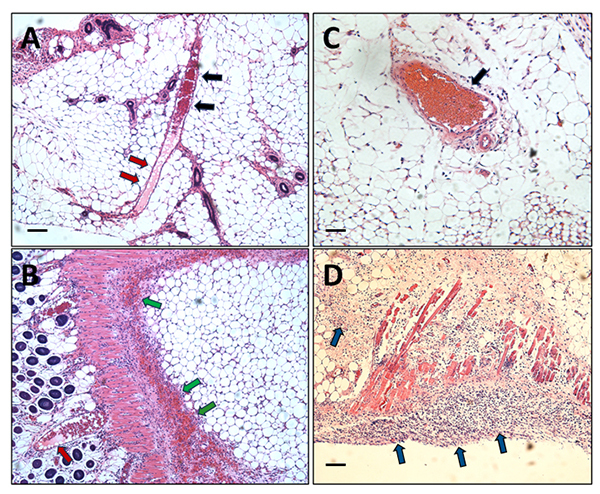
Red blood cell aggregation, presence of fibrinoid-like material in blood vessels, and inflammatory infiltrate in the skin of mice injected with fraction 4 and purified ~34 kDa GAPR1-like protein. **(A)** Skin of mice injected i.d. with fraction 4 showing capillary erythrocyte aggregation (black arrows), a hyaline material inside (red arrows), and **(B)** extensive hemorrhage (green arrows). **(C)** Skin of mice injected i.d. with purified GAPR1-like protein, showing the same pattern of red blood cell aggregation (black arrow) and **(D)** the presence of significant leukocyte infiltration (blue arrows). Scale bars represent 50 µm.

Since fraction 4 (and not fraction 2, which contains the cytolysins), was the one inducing the skin injury (bleeding and inflammation, Figures 6 A  and 6B), we purified the main protein present to try to reproduce the *in vivo* effects in mice. Even when we did not see a lesion at the macroscopic level, histological analysis showed a strong inflammatory reaction with leukocyte infiltrate, and it was evident the pattern of erythrocyte aggregation (Figures 6 C  and 6D). Some red blood cells appeared outside the vessels, as indicative of some level of tissue damage and hemorrhage, an effect that was much more severe when fraction 4 was injected into mice (Figure 6 B ).

**Figure 7.**
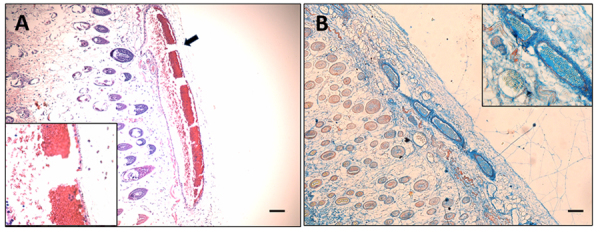
Skin of mice injected i.d. with fraction 3 and stained for the presence of fibrin with Martius, Scarlet, and Blue staining (MSB). **(A)** A clear pattern of red blood cell aggregation is observed in the hematoxylin and eosin staining and the rupture of the blood vessel basal membrane (black arrow) and erythrocyte extravasation. The inset shows a higher-power picture of red blood cell agglutination towards the capillary wall. Also, the morphology of the erythrocytes looks altered. **(B)** A hyaline substance present in the vessels stains blue (not red as expected for new fibrin deposits). Erythrocytes are stained in yellow color. Insets show an amplification of the same blood vessels. Blue staining of the hyaline material (normally observed for collagen staining) could correspond to pseudo-collagen materials, such as mature fibrin or amyloids. Scale bars represent 50 µm.

Fraction 3 also caused red blood cell aggregation towards the vessel walls and basal membrane rupture. Erythrocytes look adhered to each other, forming a package, and bound to the endothelial cells of the microvasculature (Figure 7 A ).

### Skin tissue staining for the presence of fibrin

A fibrinoid-like material was observed in some blood vessels of mice injected with fractions 3 and 4, and to corroborate whether these deposits were made of fibrin, we stained the histological slides containing the skin sections with a fibrin-specific Martius, Scarlet, and Blue (MSB) staining procedure. No significant evidence of red coloration was observed, indicating that this hyaline substance could probably be made of something else than fibrin (Figure 7 B). Interestingly, the fibrinoid-like material stained blue as expected for collagen, something attributed to amyloids and mature fibrin, which has been shown could display pseudo-collagen characteristics when becoming hyalin substances [42,43]. Amyloids also react to MSB staining and under the fixative conditions of our histological protocol, they could display a blue coloration [44]. Preliminary results suggest that the observed substance probably does not show amyloid-like properties, since it is not stained with Congo Red (not shown). 

### *In vitro* fibrinogenolytic activity induced by lionfish venom

**Figure 8.**
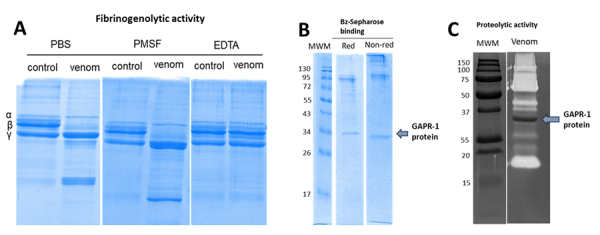
*In vitro* fibrinogenolytic activity induced by lionfish venom and ~34 kDa GAPR1-like protein characterization of potential proteolytic activity. **(A)** Fibrinogen was incubated with the extract for 3 hours in the absence or presence of two protease inhibitors (PMSF and EDTA). PMSF acts as a serine proteinase inhibitor, and EDTA is a cation-chelating agent that inhibits metalloproteinases. Fibrinogen β-chain is completely degraded, and the effect was only inhibited by EDTA. **(B)** Venom was separated by affinity chromatography in a Benzamidine-Sepharose column evidencing that, like serine proteases, GAPR1-like protein displays affinity to benzamidine. **(C)** Zymography using gelatin as a substrate to determine proteolytic activity of GAPR1-like protein, showing negative results.

To follow up on the characterization of the composition of the blood vessel fibrinoid substance observed in the skin of mice injected with fractions 3 and 4, and since mature fibrin could stain blue under the MSB procedure, we tested the activity of the extract on fibrinogen, which could suggest *in vivo* fibrin formation. When fibrinogen was incubated with lionfish venom for 3 hours, the β-chain was degraded, and the effect was inhibited by EDTA, which demonstrates that the *in vitro* effect is probably due to metalloproteinases (Figure 8 A ). However*, in vitro* pro-coagulation activity of human plasma was not observed when incubated with lionfish venom (data not shown), which suggests that hyaline material deposits on blood vessels are probably not formed by fibrin.

### Characterization of ~34 kDa GAPR1-like protein

We were able to purify the GAPR1-like protein of ~34 kDa from fraction 4 and the crude venom extract, by using affinity chromatography in a Benzamidine-Sepharose column. The rationale behind using this affinity-chromatography approach was that some GAPR1-like proteins displayed proteolytic activity [45, 46], and this type of affinity chromatography is used for the purification of serine proteases. 

The main protein in fraction 4 showed affinity to benzamidine, like other putative higher molecular weight proteases, possibly corresponding to serine proteinases present in the venom extract (Figure 8 B ). However, no proteolytic activity was displayed by the protein when tested by gelatin zymography (Figure 8 C), indicating that proteolytic activity is not related to the hemorrhagic effects previously observed in mice skin when this protein was injected.

## Discussion

Several transcriptomic and proteomic analyses have demonstrated that only a few proteins are present in the spine venoms of Scorpaeniformes, including the pore-forming toxins or cytolysins, hyaluronidases, and some lectins [24-26, 29]. In *Synanceia horrida* (stonefish) venom, Ziegman et al. [15] included the presence of what they called other “accessory molecules”, not considering them as toxins but as putative contributors to the adverse effects of the envenomation. This terminology is somehow confusing since some of these so-called accessory molecules could be components without currently assigned toxicological effects, such as some of the ones we focused on in this study. 

In the lionfish venom, in addition to the previously reported cytolysins and hyaluronidases [26, 29], we observed the presence of an abundant protein, belonging to the cysteine-rich secretory proteins, antigen 5, and pathogenesis-related 1 protein (CAP) superfamily. The identified sequences show similarity to the sperm-coating glycoprotein (SCP) domain from the Golgi-associated plant pathogenesis-related protein-1 (GAPR1). This family also includes cysteine-rich secretory proteins (CRISPs), ubiquitous proteins present in several venomous secretions of vertebrates and invertebrates. Also, some allergens found in hymenopteran sting secretions belong to this family of proteins [45, 46]. Functions associated with these venom proteins include hemorrhage, edema, neurotoxicity, immune responses, increase in vascular permeability, alterations in blood coagulation, platelet aggregation, and hemostasis impairment [47]. In addition, their upregulation is part of the response of several organisms to stress [48].

The lionfish main SCP-domain containing protein is a ~34 kDa molecule, identified by mass spectrometry from SDS-PAGE gels of size-exclusion HPLC and RP-HPLC fractions. Moreover, in this venom, we identified a ~15 kDa protein with the same partial sequences (obtained by mass spectrophotometry), present in the ~34 kDa GAPR1-like protein (Table 1). These two proteins were the main components present in the size-exclusion HPLC fractions 4 and 5 of our study. Since it was described that CAP domain proteins could form dimers [49], a possibility was that we could be in the presence of the same protein, a dimer, and a monomer. However, SDS-PAGE under reducing and non-reducing conditions showed the same mass in the proteins of these two fractions, which together with the denaturing effect induced by the SDS detergent, would rule out the dimer hypothesis. Other possibilities could be that the ~34 kDa GAPR1-like protein would contain more than one CAP domain in its primary sequence or an additional amino acid residue extension to the CAP domain present in the smaller molecule. The fact that the mass is almost double suggests the possibility of being in the presence of a two-CAP domain protein. This has been previously reported for another GAPR1, the one secreted by the infectious stage of the hookworm *Necator americanus*. This organism expresses two CAP-domain proteins, Na-ASP-1, a molecule with two CAP domains, and a single-CAP domain protein named Na-ASP-2 [50]. 

**Table 1.** Lionfish venom GAPR1-like protein sequences identified in this analysis compared to the sequences from other GAPR1-like proteins from teleost fish, including stonefish venom. In the bottom part of the table, CAP motifs from two of the sequences are highlighted in different colors for better identification. UniProt accession numbers are included as references.

***Pterois volitans* (one and two CAP domain-proteins?)**	***Synanceia horrida* (one CAP domain-protein), ref. [15]**	***Sander lucioperca* (three CAP domain-protein), A0A8D0ASN6**	***Gasterosteus aculeatus* (one CAP domain-protein), G3NGY5**
DASFQQEFLET**HNAYR** **(CAP3 motif)**	NASFQQEFLETHNNYR	DANFQREFLETHNAYR DASFQQEFLETHNAYR YASFQREFLETHNAYR	DASFQEEFLETHNAYR
GKEAVDSWYSEIK	GKEPVDTWYNEIN	GKEAVDSWYREIK GKEAVDSWYSEIK GKEAVDSWYSEIK	GKEAVDSWYSEIK
SNT**GHFTQVVWKES** FNT**GHFTQVVWKES** **(CAP1 motif)**	SNTGHFTQVVWKES	SNTGHFTQVVWKDS SNTGHFTQVVWKDS SNTGHFTQVVWKDS	SDTGHFTQVVWKDS
TELGVGMATDGRRVF**VVGQYRPA** **(CAP2 motif)**	TELGVGMATDGRRVFVVGQYRPA	KELGVGMATDGHKVFVVGQYRPA KELGVGMATDGHKVFVVGQYRPA KELGVGMATDGHKVFVVGQYRPA	TELGVGMATDGRRVFVVGQYRPA
**SCP domain-containing protein (GAPR1), *Synanceia horrida* (stonefish, venom)** MGVCLLLPRRRGRGRAEIYKYQHRDSRRNCIFTGHPSRPRASLHTVPMANASFQQEFLETHNNYRAKHNSPPMTLNSKMSASAQKWAEHLLAINTLMHRDYQARDHDGENIYCMSGTSTITLTGKEPVDTWYNEINNYNWGYPGFRSNTGHFTQVVWKES TELGVGMATDGRRVFVVGQYRPAGNMNMPGHFERNVLRLA			
**SCP domain-containing protein (GAPR1), *Gasterosteus aculeatus* (three-spined stickleback)** MADASFQEEFLETHNAYRAKHNTPKMTLNQELTASAQKWADQLLATNTMQHSETADGENIYGMSSSAPIKPTGKEAVDSWYSEIKDYKWSSPGFQSDTGHFTQVVWKDS TELGVGLATDGKRVFVVGQYRPAGNMNMPGYFEKNVCPLA			

Another interesting fact about lionfish ~34 kDa GAPR1 is that it binds to benzamidine-coupled Sepharose beads. Benzamidine is a molecule utilized as an inhibitor of serine proteases that, coupled to Sepharose, is commonly used for their purification. The catalytic triad formed in the active site of the dimer state of these enzymes interacts with benzamidine [51]. However, so far only one protein belonging to the CAP-domain family, the cone snail Tex31, has been shown to display proteolytic activity [45, 46]. Lionfish ~34 kDa GAPR1, on the other hand, did not show this activity on gelatin, which rules out the possibility of being a protease such as Tex31, unless its substrate specificity differs.

Fractions 4 and 5, which contain this type of protein, and the purified ~34 kDa GAPR1 resulted in toxicity to mice, by inducing inflammatory reactions and, in the case of these fractions, hemorrhagic injuries on the skin. Ziegman et al. [15] previously reported the presence of a GAPR1 in the venom of stonefish *Synanceia horrida*, with a sequence that contains a CAP-domain and an additional N-terminal 45-amino acid residue-long domain, not found in other members of the GAPR1 group, such as in the human version and some non-venomous teleost fish GAPR1-like proteins (Table 1). Although its function is unknown, these authors suggested a possible role of stonefish GAPR1 protein in the inflammation induced by this venom in different organs.

The presence of GAPR1-like proteins in non-venomous fish suggests that they could be skin ichthyocrionotoxins, however, our preliminary results indicate that they are found in *P. volitans* dorsal spine extracts and not significantly expressed in epidermal mucus (not shown). In any case, lionfish spine toxic secretion is a combination of venom and mucus components, and in our toxicological study, we did not differentiate between both origins.

Interestingly, there is evidence that the CAP-domain protein family could form Zn^+2^-dependant amyloid aggregates [51]. Amyloid fibrils are often toxic to cells since they participate in inflammatory processes, which could cause tissue injury. Interestingly, GAPR-1 proteins require the assembly into amyloids to be toxic, as was observed for natrin, a CRISP present in cobra *Naja atra* venom, that acts as an inflammatory modulator by affecting endothelial cell adhesion receptors [12, 52]. Moreover, CRISPs from rattlesnake venoms can induce acute inflammatory responses and increase vascular permeability in different organs of animal models. It has been shown that these venom CRISPs promote the activation of leukocytes, the release of several inflammatory mediators, and the activation of an innate immunity response [53]. 

There are four identified CAP motifs characteristic of this family, displaying some amino acid variations, and some of them are predicted to mediate the formation of amyloid fibrils [54]. CAP-1 is a predicted amyloid motif that we identified in the sequence of lionfish venom GAPR1 proteins. The sequence motif is GHFTQVVWKES. This strongly suggests that amyloid fibrils could be potentially forming in mice injected with this venom and fractions. In the blood vessels of mice injected with these fractions, we observed the presence of a fibrinoid-like material, that is not significantly recognized by fibrin-specific stains. Even when this substance does not seem to react to human amyloid stains (preliminary results), these deposits could be formed by GAPR1-like amyloid substances, and perhaps be involved in the inflammation-induced toxicity caused by lionfish venom. This type of material could also alter red blood cells and provoke their aggregation and binding to endothelial cells, an event observed in the blood vessels of mice injected with this protein and fractions 3 and 4. The presence of amyloid fibrils could cause a similar red blood cell effect, an event that has been reported in human vascular amyloidosis [55]. 

Regarding the presence of these hyaline deposits on blood vessels, an interesting medical case of a recent accident caused by a blackbelly rosefish (*Helicolenus dactylopterus*)*,* a member of the fish Scorpaenidae family, reported a patient with skin lesions like the ones observed in leukocytoclastic vasculitis, an inflammatory pathology characterized by the presence of fibrinoid necrosis of small vessels [56]. This vasculitis is a hypersensitivity neutrophil-recruiting reaction against immune complex deposits associated with the walls of blood vessels, resulting in inflammatory tissue damage. A similar mechanism could be occurring in the case of envenomation by other Scorpaeniforms, such as lionfish since some similar histopathological characteristics were observed in our mice experimental model. 

On the other hand, hyaluronidase-rich fraction 3 was one of the most damaging for the skin, both macroscopically and at the microscopic level. Degrading hyaluronic acid probably alters the integrity of the extracellular matrix causing the rupture of the vessel wall, as has been suggested in envenomation by other organisms [33]. This effect combined with the one induced by GAPR1, could result in damage to the skin and the observed hemorrhagic lesion. 

Interestingly, in gastrocnemius muscle, lionfish venom was able to induce severe myonecrosis and hemorrhage after 24 hours, an effect that was not observed at earlier times [23]. However, according to our results, venom fraction 1, which induces the significant myonecrotic effect, did not seem to cause hemorrhage, an effect that, as we explained above, was induced by other venom compounds (present in fractions 3-5). In addition, in another study [23], no direct cytotoxicity was observed on myoblasts, a characteristic event induced by myonecrotic fish venoms such as the one from *Thalassoprhyne nattereri* [57]. 

At this point, we do not know what toxins could be responsible for the observed myonecrotic effect. Some proteases have been identified in the extract, most of them probably derived from epidermal mucus, which could indirectly participate in the pathological process. These include putative aminopeptidases, peptidase M3, thimet oligopeptidases, aspartyl aminopeptidases, peptidase S1, prolyl endopeptidases and calcium-activated neutral peptidase 1, among others (not shown). However, it seems that no significant hemorrhage is caused by this fraction, then the possibility of ischemia-induced gastrocnemius myonecrosis caused by bleeding seems unlikely. Other causes of ischemia, such as blood flow obstruction due to clot formation, for instance, could be associated with muscle damage and proteases present in lionfish venom/mucus could be affecting blood coagulation. However, even when *in vitro* fibrinogenolysis is observed by incubation of fibrinogen with the venom extract, no direct *in vitro* pro-coagulant events were observed on human plasma (not shown). This does not rule out a possible *in vivo* event due to endothelial cell alterations, which could cause blood clotting as has been suggested for the thrombi formation induced by *Thalassoprhyne nattereri* envenomation [57].

In envenomation by other types of fish as the *Potamotrygon henley* stingray, human cardiomyocytes were altered and the effect has been associated with the inflammation *per se*, specifically as a neutrophil-mediated injury [58]. The strong inflammatory reaction observed in lionfish-injected mice tissues suggests that gastrocnemius damage could be caused directly by the inflammatory infiltrate, but more investigation is required to confirm this hypothesis.

An intriguing result from this study was that fraction 2, the one containing the cytolysins/hemolysins supposedly responsible for the toxic effects of this kind of fish, was not associated with the myonecrotic and hemorrhagic activities. The presence of these cytolysins was confirmed by proteomic analysis and the hemolytic activity on rabbit erythrocytes was corroborated during the experimental procedures. Through proteomics, we identified almost the complete sequences of proteins with high similarity to Pv toxins α and β from *Pterois volitans*, *Pterois lunulata,* and *Pterois antennata*, and Tx α, β and ϒ from *Dendrochirus zebra* (results not shown). The only evident effect observed in mice tissue injected with this fraction was the presence of inflammatory infiltrate, but we cannot confirm that inflammation was induced by these pore-forming toxins, since it could be due to other components present in this fraction of the venom.

Compared to the venoms of other vertebrate organisms, the characterization of fish venoms has been relegated in terms of their composition and mechanisms of action, probably because most of their components do not induce direct tissue damage but, instead, display indirect cytotoxic activities, which are only evident *in vivo*. That is the reason why, *in vitro*, most fish venoms have not shown clear and definitive toxic effects. In this *in vivo* experimental model, we evidenced that lionfish venom can induce severe myonecrosis and prominent lesions in the skin in 24 hours, characterized by microvasculature alterations and hemorrhage. These injuries are, without a doubt, at least partially caused by the inflammatory response that leads to tissue damage and excruciating pain. We consider that from now on, attention should be focused on new potentially toxic compounds, such as the CAP-domain proteins and hyaluronidases, most of which have, so far, been minimized, dismissed, or even considered just secondary contributors to the envenoming. The study of these molecules could open a whole new scenario of mechanistic possibilities of inducing damage to tissues and organs. 

## Conclusions

Lionfish dorsal spine venom, combined with epidermal mucus components, induces 24-hour toxicity in mice gastrocnemius muscle and skin. When venom was separated by size-exclusion HPLC, we observed that some of the fractions were able to induce *in vivo* injuries that included myotoxicity and bleeding. The lionfish cytolysin-containing fraction did not induce *in vivo* toxicity in mice, only some inflammation. Other compounds identified in the venom were hyaluronidase and GAPR1-like proteins, in fractions able to induce microvascular alterations and injury, and which, associated with the evident leukocyte infiltration and blood extravasation, would result in a macroscopic hemorrhagic lesion to the skin. 

## Availability of data and materials

 The data that support the findings of this study are available from the corresponding author, upon reasonable request.
